# Prophylactic Antibiotics in Otolaryngologic Surgeries: From Knowledge to Practice

**Published:** 2012

**Authors:** Minoo Khatami-Moghadam, Mohammad-Taghi Khorsandi-Ashtiani, Mohammad-Ali Mohagheghi, Mehrdad Hasibi, Ali Kouhi

**Affiliations:** 1 *Otorhinolaryngology Research Center, Tehran University of Medical Sciences, Amir-Alam University Hospital*; 2 *Cancer Institute, Cancer Research Center, Tehran University of Medical Sciences*; 3 *Department of Infectious Diseases Tehran University of Medical Sciences, Tehran, Iran*

**Keywords:** Antibiotic, Head, Neck, Otolaryngology, Prophylaxis

## Abstract

**Introduction:**

The management and use of antimicrobial drugs has clinical, economic, and environmental implications. In many countries, antimicrobial drugs are the most frequently prescribed therapeutic agents. Therefore, health-care policy should focus on how to establish a rational attitude toward antibiotics. This study was performed to investigate antibiotic usage as a prophylactic regimen in head and neck surgeries.

**Materials and Methods:**

This study was a retrospective case series. Patients undergoing otolaryngology surgeries in a tertiary referral otolaryngology center were included. Members of operating room staff that were unaware of the study objectives collected patients’ data using a questionnaire that contained information regarding general medical condition, disease, surgical procedure, and prophylaxis regimen and duration.

**Results:**

Excluding infected patients, we studied 1349 patients during a four-month period who needed prophylactic antibiotics. A total of 34 different types of surgical procedures were performed. Out of the total number of patients, 503 (37.0%) received a parenteral antibiotic directly before surgery. The main antibiotics used before surgery were cephalosporins (94.9%). All of the 1349 patients were administered antibiotics after the procedure. These antibiotics where given with a mean number of doses of 4.81 (range: 1–68), and also consisted of mostly cephalosporins.

**Conclusion:**

Our results indicate that prophylactic antibiotics were being significantly misused in a tertiary referral center of a university hospital. Although teaching the principles of prophylaxis to physicians is important, we think that finding a way to bring this knowledge to practice is more important.

## Introduction

The WHO conference on the rational use of drugs in 1985 marked the beginning of efforts to improve the use of drugs, especially in developing countries (1). In 1993, the WHO Action Program on Essential Drugs (WHO/DAP) published the manual *How to investigate drug use in health facilities *in response to the increased awareness of the problems impeding the rational use of drugs (2). Now, we clearly know that the management and use of antimicrobial drugs has clinical, economic, and environmental implications. In many countries, antimicrobial drugs are the most frequently prescribed therapeutic agents, accounting for 30 to 50 percent of drug prescriptions (3). In economic terms, expenditures on antimicrobial drugs in the year 2000 were projected to be $40 billion (4). In addition, the highest levels of resistance to antimicrobial drugs occur in countries with the highest levels of antimicrobial drug use (5). It has been estimated that hospital-acquired infections due to drug-resistant organisms cost the United States $4 billion in 1990 (6). 

The high cost of treating drug-resistant infections may exceed the financial capacity of many patients and hospitals in developing countries. Thus, managers must monitor and minimize antibacterial resistance in their hospitals. Moreover, the use of antibiotics may encourage laxity of good surgical technique and promote the occurrence of super-infections. Antibiotic use is also associated with allergic reactions, toxic reactions, and adverse effects. Therefore, health care policy should focus on how to establish a rational attitude toward antibiotics. Short-term antibiotic prophylaxis (24 or 48 hours) is as efficient as long-term (7). A safe reduction in the use of antibiotics can be based only on solid comparative studies with evidence authoritative enough to be able to convince not only academic researchers but also the physician "in the field" (8). Due to a lack of consensus, physicians’ practices are different and can even be different from the literature and their own personal beliefs (9). This study was performed in order to investigate antibiotic usage as a prophylactic regimen in head and neck surgeries.

## Materials and Methods

In this retrospective case series, patients undergoing otolaryngology surgeries in a tertiary referral otolaryngology center in Iran were included during four months of the year (second month in each season). Patients with a medical history of previous surgery (increased risk of infection due to scarring), radiation injury (decreased host defenses), and certain medical conditions, such as diabetes mellitus or HIV (predisposes the patient to infection), were excluded from the study. Infected surgical procedures and antibiotic usage for infected wounds that did not meet the prophylaxis definition were also excluded. Study indicators were selected according to the US Agency for International Development’s working draft (3). The main reference for comparing our results was the antibiotic prophylaxis guidelines of the American Academy of Otolaryngology (10).

Members of the operating room staff that were unaware of the study objectives collected patients’ data using a questionnaire, which contained information regarding the patient’s general medical condition, disease, surgical procedure, and prophylaxis regimen and duration. The attending physicians were unaware of the study and data collection as well. In order to compare the actual administration of antibiotics with physicians’ knowledge of prophylactic antibiotics, we asked physicians to answer a questionnaire regarding antibiotic prophylaxis in otolaryngology procedures. It should be mentioned that physicians were unaware of this study. All of the attending otolaryngology physicians filled in the questionnaire (Table 4). The form contained questions about whether they should prescribe prophylactic antibiotics for a specific procedure or not and if they should, what type of antibiotic should be used and should it be prescribed pre-operatively, post-operatively, or after discharge. The percentages of positive answers are presented in Table 4. 

The Statistical Package for Social Sciences (SPSS; version 16) was used for data analysis.

## Results

**Antibiotic usage data:** During the four-month period of the study 1433 operations were done in our center. There were 766 male (median age: 25 years; range: 1–88) and 667 female (median age: 26 years; range: 1–84) patients. Excluding infected patients, we studied 1349 patients needing prophylactic antibiotics. A total of 34 different types of surgical procedures were performed (Table 1). 

**Table 1:** Numbers of each surgical procedure performed.

**Type of surgery**	**Frequency**	**Percent (%)**	**Type of surgery**	**Frequency**	**Percent (%)**
VT	14	1.0	Middle ear exploration	81	6.0
Cochlear implant	15	1.1	FESS	83	6.2
Canaloplasty	15	1.1	Septoplasty	110	8.2
Cleft lip	15	1.1	Nasal fracture	143	10.6
Mandible fracture	16	1.2	Adenotonsilectomy	150	11.1
Parotidectomy	33	2.4	Aerodigestive endoscopy	167	12.4
Neck mass	37	2.7	Tympanomastoidectomy	363	26.9

VT: ventilation tube administration; FESS: Functional endoscopic sinus surgery.

The most prevalent procedures (those with frequency of more than 1%) were studied regarding the antibiotic regimen used. Descriptive data for antibiotic use in these procedures is given in Table 2. 

**Table 2:** Antibiotic regimen for common procedures

**Procedure**	**No**	Prophylaxy ab*	**PO route** **(Oral/ parenteral)** ^+^	**PO dose**	**Discharge ab** ^§^	**Duration of home ab (day)** ^ §§^
**VT**	14	2 (14.3%)	9/5	4.36 (3-8)	14 (100%)	5.43 (5-7)
**Cochlear implant**	15	5 (33.3%)	0/15	5.20 (2-9)	15 (100%)	7.13 (5-10)
**Canaloplasty**	15	3 (20.0%)	3/12	3.60 (3-4)	15 (100%)	6.20 (4-10)
**Cleft lip**	15	1 (6.7%)	3/12	6.93 (3-16)	15 (100%)	6.53 (5-14)
**Mandible fracture**	16	5 (31.2%)	0/16	7.00 (3-20)	16 (100%)	6.25 (5-10)
**Parotidectomy**	33	11 (33.3%)	1/32	11.15 (3-48)	32 (97%)	5.76 (0-10)
**Neck mass**	37	5 (13.5%)	5/32	6.62 (2-24)	37 (100%)	6.76 (5-15)
**Middle ear exploration**	81	71 (87.7%)	4/77	3.85 (1-16)	81 (100%)	5.85 (3-10)
**FESS**	83	16 (19.3%)	2/81	6.52 (2-32)	81 (97.6%)	6.01 (0-10)
**Septoplasty**	110	4 (3.6%)	14/96	3.78 (3-5)	109 (99.1%)	5.95 (0-10)
**Nasal fracture**	143	10 (7.0%)	15/128	3.39 (3-5)	143 (100%)	5.34 (5-7)
**Adenotonsilectomy**	150	0 (0.0%)	14/136	3.25 (3-5)	150 (100%)	5.18 (3-10)
**Aerodigestive endoscopy**	167	20 (12.0%)	41/126	6.38 (1-48)	162 (97%)	5.43(0-10)
**Tympanomastoidectomy**	363	343 (94.5%)	5/358	3.62 (1-16)	361 (99.4%)	6.02 (1-30)

number of procedures where patients received antibiotics within half an hour of the procedure beginning; +type of post-op antibiotic regimen, whether oral or parenteral; ^§^number of patients receiving antibiotics at discharge; ^§§^ number of days of antibiotic treatment after discharge

PO: post-operative; ab: antibiotic; VT: ventilation-tube; FESS: functional endoscopic sinus surgery

Out of the total patient group, 503 patients (37.0%) received parenteral antibiotics directly before surgery. The main antibiotics used were cephalosporins (94.9%). All of the 1349 patients were administered antibiotics after a procedure (parenteral: 985 [73.0%], oral 364 [27.0%]). These antibiotics where given with a mean number of doses of 4.81 (range: 1–68), and they consisted of mostly cephalosporins (Table 3). 

**Table 3:** Different types of antibiotics given post-operatively

**Drug**	**Frequency**	**Percent**
Ceftazidime	1	0.1
Ciprofloxacin	1	0.1
Coamoxiclav (syrp)	1	0.1
Cefazolin / Cefixime	1	0.1
Ceftriaxone / Ampicillin	1	0.1
Metronidazole / Clindamycin	1	0.1
Cephalexin / Metronidazole	1	0.1
Cefazolin / Ciprofloxacin	1	0.1
Cefixime	2	0.1
Penicillin / Metronidazole	4	0.3
Ampicillin (syrp)	4	0.3
Clindamycin (amp)	4	0.3
Ampicillin (amp)	7	0.5
Cefazolin / Ceftriaxone	7	0.5
Ceftriaxone / Clindamycin	12	0.9
Cefazolin / Metronidazole	20	1.5
Ceftriaxone	36	2.7
Cephalexin	356	26.4
Cefazolin	888	65.8
Total	1349	100.0

Only 15 patients were antibiotic-free on discharge (99.0% still taking antibiotics on discharge). The mean duration of outpatient treatment with antibiotics was 5.86 days (range: 0–30).

**Economic calculations: **Comparison of our results with the American Academy guidelines shows that most of the antibiotic regimens prescribed could be defined as over-treatment. Calculation of the extraneous antibiotic usage (i.e. the amount of antibiotic that should not have been administered) revealed that more than 37,000 grams of oral and 13,000 grams of intravenous preparation of 1^st^ generation cephalosporins were used inappropriately. The estimated cost of the excess antibiotics was approximately 127,462, 000 Rls.

## Discussion

The use of prophylactic antibiotics aims to provide effective levels of drug at the time of wound exposure (3). Prophylaxis is meant to augment host defenses at the time of bacterial invasion, and is an attempt to attack organisms before any infection has occurred. The regimen is mainly directed against the most common infectious microbes. According to the available protocols, many procedures require a single dose given before wound exposure to provide adequate tissue concentration throughout the operation (2, 3). In certain circumstances, such as prolonged surgery that lasts for more than 4 hours and massive blood loss, additional doses should be administered.

Different protocols have been provided regarding types of surgery and fields exposed during operation; for example, if the mucosa are not penetrated, only gram-positive skin flora should be targeted, but if the mucosa are affected then post-operative anaerobic infection should be assumed and an appropriate wide-spectrum antibiotic should be administered (1, 2).

Prophylactic antibiotic regimens are changing day to day to decrease associated problems such as microbial resistance, expenses, adverse effects, duration of hospital admission, and so on (6, 10). This is why lots of efforts have been made to establish these rules globally. All of the guidelines, protocols, and conferences are held to teach physicians how to improve their practice in this regard. Efficacy of these regimens is proven by the available evidence. But the question is whether physicians transfer this knowledge to their practice or not?

According to the American Society of Health system Pharmacists (ASHP) guidelines, antimicrobial prophylaxis should be (i) active against likely microbial contaminants, (ii) given in an appropriate dosage and at a time that ensures adequate concentrations at the incision site during the period of potential contamination, (iii) safe, and (iv) administered for the shortest effective period to minimize adverse effects, development of resistance, and cost (11). 

These recommendations are in contrast to the use of prophylactic antibiotics that we observed in our study. Our results indicate a significant level of incorrect use of prophylactic antibiotics in a tertiary referral center of a university hospital. The important point is that all of the attending physicians knew the right thing to do, and had knowledge of the right antimicrobial regimens, but there was a great discrepancy between theory and practice. To study this effect we asked the attending physicians to fill in a form regarding their knowledge about antibiotic regimens; Table 4 shows this data. 

**Table 4:** Physician’s knowledge regarding prophylaxis regimens. Comparison of these data with Table 2 shows discrepancies between practice and knowledge.

**Type of surgery**	**Prophylaxis** ^1^	**Type of antibiotic** ^2^	**Pre-OP** ^3^	**Post-OP** ^3^	**Discharge** ^3^
VT	37.5%	Cephalosporin	37.5%	25%	25%
Cochlear implant	62.5%	Cephalosporin	62.5%	37.5%	12.5%
Canaloplasty	50%	Cephalosporin/Ciprofloxacin	50%	37.5%	25%
Cleft lip	50%	Cephalosporin/Ampicillin	50%	37.5%	12.5%
Mandible fracture	62.5%	Cephalosporin	62.5%	50%	37.5%
Parotidectomy	37.5%	Cephalosporin	37.5%	12.5%	12.5%
Neck mass	37.5%	Cephalosporin	37.5%	25%	0%
Middle ear explore	37.5%	Cephalosporin	37.5%	25%	25%
FESS	50%	Cephalosporin	50%	37.5%	25%
Septoplasty	37.5%	Cephalosporin	37.5%	25%	25%
Nasal fracture	12.5%	Cephalosporin	12.5%	12.5%	12.5%
Adenotonsilectomy	37.5%	Cephalosporin/Ampicillin	37.5%	25%	12.5%
Aerodigestive endoscopy	12.5%	Ampicillin	12.5%	0%	0%
Tympano-mastoidectomy	50%	Cephalosporin	50%	37.5%	25%

1, Percent of attending physicians who stated they would prescribe prophylatic antibiotics for the procedure; 2, The most common antibiotic family physicians stated should be prescribed; 3, Percent of physicians who indicated they would prescribe antibiotics pre-operatively, post-operatively, or after discharge.

Although part of the discrepancy may be due to the fact that most of the antibiotics were prescribed by the residents and not directly by attending physicians, the data clearly shows the difference between knowledge and practice, as the attending physicians can supervise and monitor residents if they believe the treatment given is wrong. Although teaching the principles of prophylaxis to the physicians is important, we think that finding a way to bring this knowledge into practice is more important. As our economic calculations show, the positive effects of correcting this malpractice are obvious, and any effort in this regard has economic rationales.

In a similar study by Fennessy and colleagues in Ireland, clinicians’ habits regarding antimicrobial prophylaxis in otolaryngology was studied. The results of the study indicated that 42% of patients who ought to have received prophylaxis did not, while 46% of those who did not require it received it. 

In their patient group the timing of antibiotic treatment was unsuitable in 41% of cases. Like ours, this study shows evidence of the unnecessary administration of antimicrobial agents perioperatively and the presence of subclinical intraoperative antimicrobial levels for prophylaxis in many otorhinolaryngologic operations. Therefore, it seems that this problem exists in many settings. According to the WHO, it is the responsibility of the hospitals to control and improve the prescribing practices of physicians (2) but it seems that more effective ways should be found.

## Conclusion

 Pitfalls in the practice of administering prophylactic antibiotics have always been present. The main attempts to resolve this problem have been to increase the information and knowledge available to physicians. Our results show that although teaching the principles of prophylaxis to physicians is important, even physicians with good knowledge of how to prescribe prophylactic antibiotics are over-treating patients in their actual practice. Finding a way to translate knowledge into practice is more important and should be the subject of future training in this field.
